# Correlation between hospital finances and quality and safety of patient care

**DOI:** 10.1371/journal.pone.0219124

**Published:** 2019-08-16

**Authors:** Dean D. Akinleye, Louise-Anne McNutt, Victoria Lazariu, Colleen C. McLaughlin

**Affiliations:** 1 School of Public Health, State University of New York, University at Albany, Albany, NY, United States of America; 2 Institute for Health and the Environment, State University of New York, University at Albany, Albany, NY, United States of America; 3 Albany College of Pharmacy and Health Sciences, Albany, NY, United States of America; Yokohama City University, JAPAN

## Abstract

**Background:**

Hospitals under financial pressure may struggle to maintain quality and patient safety and have worse patient outcomes relative to well-resourced hospitals. Poor predictive validity may explain why previous studies on the association between finances and quality/safety have been equivocal. This manuscript employs principal component analysis to produce robust measures of both financial status and quality/safety of care, to assess our *a priori* hypothesis: hospital financial performance is associated with the provision of quality care, as measured by quality and safety processes, patient outcomes, and patient centered care.

**Methods:**

This 2014 cross-sectional study investigated hospital financial condition and hospital quality and safety at acute care hospitals. The hospital financial data from the Centers for Medicare and Medicaid Services (CMS) cost report were used to develop a composite financial performance score using principal component analysis. Hospital quality and patient safety were measured with a composite quality/safety performance score derived from principal component analysis, utilizing a range of established quality and safety indicators including: risk-standardized inpatient mortality, 30-day mortality, 30-day readmissions for select conditions, patient safety indicators from inpatient admissions, process of care chart reviews, CMS value-based purchasing total performance score and patient experience of care surveys. The correlation between the composite financial performance score and the composite quality/safety performance score was calculated using linear regression adjusting for hospital characteristics.

**Results:**

Among the 108 New York State acute care facilities for which data were available, there is a clear relationship between hospital financial performance and hospital quality/safety performance score (standardized correlation coefficient 0.34, p<0.001). The composite financial performance score is also positively associated with the CMS Value Based Purchasing Total Performance Score (standardized correlation coefficient 0.277, p = 0.002); while it is negatively associated with 30 day readmission for all outcomes (standardized correlation coefficient -0.236, p = 0.013), 30-day readmission for congestive heart failure (standardized correlation coefficient -0.23, p = 0.018), 30 day readmission for pneumonia (standardized correlation coefficient -0.209, p = 0.033), and a decrease in 30-day mortality for acute myocardial infarction (standardized correlation coefficient -0.211, p = 0.027). Used alone, operating margin and total margin are poor predictors of quality and safety outcomes.

**Conclusions:**

Strong financial performance is associated with improved patient reported experience of care, the strongest component distinguishing quality and safety. These findings suggest that financially stable hospitals are better able to maintain highly reliable systems and provide ongoing resources for quality improvement.

## Background/Introduction

Is the financial status of a hospital related to the quality and safety of care delivered? While this simple and straightforward question has attracted considerable attention, it has been remarkably difficult to answer. Efforts to control the high costs of health care in the United States presuppose hospitals can do more with less. Hospitals face considerable pressure to lower costs while maintaining quality outcomes [1, 2]. Initiatives to financially incentivize quality, such as public reporting and value-based payment (VBP), will succeed in improving population health for all only if they are designed to account for the complicated relationship between quality and facility financial stability. Otherwise, these programs run the risk of perpetuating the “rich get richer” history of the American health care system and will continue to penalize safety net hospitals and their underserved populations[3]

Prior literature suggests that some aspects of patient care may be compromised as a hospital’s financial condition declines [4–11]. Studies directly examining the correlation between financial status and quality and safety of patient care, however, have been equivocal and the findings uncertain. Lack of clear associations may be due to the poor predictive validity of the measures of finances and of quality. When considering financial performance, many financial distress models relied on specific indicators of stress, including bankruptcy and closure data, which are easier to obtain but do not represent the range of financial health [12]. Other studies used only narrow measures of hospital financial performance (e.g., operating margin), which do not capture the full range of revenue potentially available for investment in quality improvements [13]. Concerning quality performance, studies have generally focused on specific outcomes, such as mortality, or hospital readmission from conditions such as pneumonia, heart failure, or myocardial infarction [14–17]. The expansion of public reporting by the Centers for Medicare and Medicaid Services (CMS), as part of VBP, has widened the pool of measures available for quality analysis.[14, 18].

While most previous studies have used limited approaches in describing the abstract measures representing hospital financial health and quality of care, this paper considers an entire profile of financial characteristics and patient quality and safety measures. We hypothesize that robust measurement of these financial measures and quality and safety measures improve the likelihood of observing the relationship between poor financial health and inferior hospital quality of care and patient safety. We attempted to determine whether a composite financial indicator derived from a machine learning methodology (principle component analysis) would outperform already established financial indicators used in the literature in examining the correlation with quality and safety of patient care. In the next section, we present a conceptual framework and the contribution of our analysis, followed by summarization of several studies that have assessed issues relevant to the ones we are examining. This is followed by a discussion of study methods and measures and concludes with the presentation of results and policy implications.

### Conceptual framework

The hypothesis which posits that financial status and quality/safety are linked has strong construct validity. Profitable hospitals with strong cash flows can pay off debt quicker, which allows them to further invest in capital at lower costs than cash-strapped hospitals. With more capital, these facilities can make sizeable investments in clinical and administrative information technology and monitoring systems, hire better qualified staff, sustain ongoing training programs, initiate evidence-based clinical protocols and quality improvement projects, with the goal and outcome of attracting more market share and increasing profits.[19–21] Financial distress may stem from exogenous factors, such as policy changes or local economy, while it also may be attributable to internal or efficiency issues, such as inferior services or poor management.[19, 20] Given that activities to improve hospital quality and patient safety can entail substantial costs, it is presumed that hospitals facing greater financial pressure from inadequate revenues will limit quality improvements as financial performance declines.[22] A record of hospital financial losses likely will also reduce access to capital and raise the costs of borrowing, further hindering the facility.[23, 24] Previous studies support this expectation, demonstrating declines in hospital staffing, infrastructure investment and critical process of care measures, when financial pressure mounts.[25, 26] Existing literature suggests that the lack of resources prevents safety net facilities from investing in care-improvement initiatives, which can lead to higher rates of mortality and morbidity.[27, 28] These facilities have also been shown to provide costly and overpriced care due to inefficient systems and staffing, all of which have been shown to negatively impact patient care and increase length of hospital stay.[29–32]

Value-based payment initiatives are designed to provide direct return on investment (ROI) for improved outcomes, but often presumes all facilities have comparable baseline financial resources to invest in quality improvement (QI). Actions to install QI can require significant upfront resources, and often requires already having robust financial health to engage in such initiatives.[33, 34] Additionally, many VBP initiatives target specific patient groups and specific outcomes, raising concern about disparity in investment in QI among different inpatient populations. Like VBP, public reporting of quality data is intended to incentivize improvement through connecting consumer choice to quality. Public reporting has the potential to influence reputation and, in turn, affect patient perceptions, demand for hospital services, and market share.[35, 36] Despite general support for public reporting and pay for performance initiatives, critics worry that such efforts may have a deleterious effect on safety-net providers struggling with lower reimbursement rates and higher costs associated with caring for populations with greater medical complications and socioeconomic impediments [37–39]. There is concern that these initiatives negatively target safety-net hospitals with limited resources for quality improvement programs and infrastructure.[40, 41]

In this study, we aim to answer the question of whether quality and patient safety metrics are related to hospital financial performance by examining various measures of financial performance and multiple indicators of patient quality of care and patient safety. This study is unique in that we used principal component analysis to combine multiple measures into meaningful predictive models, while creating composite and robust measures that are more discriminating in detecting differences in performance across hospitals for both our independent variable of financial health and our dependent variables measuring hospital quality and patient safety.

### Literature review

When considering the financial health of hospital facilities, varying financial indicators measuring profitability, liquidity, and solvency represent significant markers of financial health; however, discerning financial health is complicated among hospitals and it is difficult to rank the numerous indicators by importance or predictive power. Additionally, individual indicators do not necessarily capture all aspects of hospital financial health, and their order of importance is unclear since varying studies cite different measures as being the most effective indication of impending fiscal problems. [19, 42–44] Limitations of past studies include utilization of financial data that focused only on specific populations (e.g. Medicare patients), the outsized influence of facilities at the extremes of financial performance, and the employment of gross metrics such as operating margin and total margin. [45–50] Using these limited approaches, several studies have found that poor hospital financial health may lead to increased negative outcomes for some publicly reported outcomes and not others. The equivocal findings are difficult to interpret because hospital margins may be misleading indicators of financial health, and negative margin or net loss are not the sole predictors of financial distress. For instance, despite positive margins, some hospitals may have insufficient liquid assets to meet all current or future obligations surrounding quality improvement. Revenues might be underestimated because of the absence of nonoperating transfers, income from grants, loan forgiveness, or other exclusions from typical accounting reports.[51] Therefore, it is important to consider a range of financial dimensions including measures surrounding liquidity, financial leverage, and physical facilities. Wider ranging financial data that better depicts a hospitals financial health are publicly reported and available at state and federal agencies. This work shows the value of looking beyond the limited measures of hospital financial health previously utilized.

Similarly, quality and safety measures included in previous studies have been limited based on 1) use of distal outcomes such as mortality; 2) including only specific patient populations such as Medicare patients; and 3), including only select conditions, most commonly heart attack (AMI), congestive heart failure (CHF), and pneumonia (PN).[14, 17, 18, 45, 49, 52] These three conditions (AMI, CHF, PN) are among the most common causes of hospitalizations for the US population overall, particularly the elderly. There is scientific evidence supporting associations between mortality and readmission for these conditions with specific clinical care processes, leading to use of these indicators to measure provision of consistent quality of care for these conditions.[53] These mortality and readmission indicators have been used as proxies for hospital quality in many prior research articles.[18, 41, 52–56]

Several quality and safety indicators are now publicly reported, including CMS Hospital Compare VBP Total Performance Score (VBP-TPS) and a Five-Star hospital rating system (https://www.medicare.gov/hospitalcompare/Data/Hospital-overall-ratings-calculation.html). These measures, however, were designed for the specific purpose of payment reform and may not be ideal for research purposes. The VBP-TPS includes cost efficiency and year-by-year quality improvement, which may introduce confounding by past financial performance. Cefalu et al. recently reported use of principal component analysis across 25 hospital quality measures, concluding that four factors representing patient experience of care, select process of care measures, and inpatient mortality demonstrated the multidimensionality of hospital quality. [57]

## Methods

### Population

The study population included general medical/surgical hospitals in New York State (NYS) that participated in the Centers for Medicare and Medicaid Services (CMS) inpatient prospective payment system. All facilities included in this study provided a broad enough range of services to ensure availability of sufficient quality of care indicators and had financial data available in 2014. These requirements lead to the exclusion of specialty hospitals, federal hospitals, and some small hospitals providing limited services (e. g. critical care hospitals). In the situation where there were multiple hospitals in the same network, ancillary facilities without independent financial information from a principal facility were excluded from the analysis. All general medical/surgical hospitals in NYS are nonprofit or government owned.

This study was approved by the NYS Department of Health (NYSDOH) Institutional Review Board (IRB). Informed consent was not required for health services research of administrative health records. Patients were not contacted.

### Measurement of hospital quality of care and patient safety

A total of 46 indicators of quality of care and patient safety were incorporated into a composite measure, covering four domains: (1) inpatient quality, (2) patient safety, (3) process of care, and (4) patient experience of care. We call this measure the composite quality/safety performance score.

The inpatient quality domain included two Inpatient Quality Indicators (IQIs) developed by the Agency for Healthcare Research and Quality (AHRQ) and endorsed by the National Quality Forum (NQF): risk adjusted heart failure mortality rate (IQI 16) and risk adjusted pneumonia mortality rate (IQI 20). The IQI mortality rates were obtained from the NYSDOH Open Data website and based on the NYS Statewide Planning and Research Cooperative System (SPARCS) inpatient discharge data for 2014 [accessioned March 27, 2018, IQI version 5.0, March 2015].[58, 59]

The patient safety domain was assessed using 11 AHRQ Patient Safety Indicators (PSIs), also based on SPARCS data from 2014 obtained from the NYSDOH Open Data website [accessioned March 27, 2018, PSI version 6.0, September 2015].[59] The domain encompassed six measures of perioperative and postoperative adverse events. These events included postoperative hip fracture (PSI 08), perioperative hemorrhage or hematoma (PSI 09), postoperative physiologic and metabolic derangements (PSI 10), postoperative respiratory failure (PSI 11), perioperative pulmonary embolism or deep vein thrombosis (PSI 12), postoperative wound dehiscence (PSI 14), pressure ulcers (PSI 03), iatrogenic pneumothorax (PSI 06), central venous catheter-related bloodstream infection (PSI 07), accidental puncture or laceration (PSI 15), and deaths among patients with low-mortality diagnoses (PSI 02). These PSIs, except for PSI 10, are either NQF endorsed or included in the NQF endorsed composite PSI.

The process of care (also known as timely and effective strategies) domain was compiled from the CMS Hospital Inpatient Quality Reporting (IQR) Program indicators derived from chart reviews [accessioned March 27, 2018, HQA 2007, year of admission = 2014]. For each hospital, 21 process of care indicators contributed to the calculation of the composite quality/safety performance score. The five process of care categories include: emergency department throughput (six indicators), preventive care (six indicators), surgical care improvement (six indicators), pneumonia care (two indicators), and stroke care (one indicator).

The Patient Experience of Care domain was assessed via the Hospital Consumer Assessment of Healthcare Providers and Systems (HCAHPS) Patient’s Perspectives of Care Survey, a nationally standardized publicly reported survey utilized for measuring patients’ perceptions of their hospital experience. [60] Eleven HCAHPS measures are publicly reported measures on the Hospital Compare website, including six composite topics, two individual items, and three global items. Composite topics include communication with doctors, communication with nurses, responsiveness of hospital staff, pain management, communication about medications, and discharge information. Individual items include cleanliness and quietness of the hospital environment, while global items include overall rating of the hospital, willingness to recommend the hospital, and care transition—patient understanding of their care at discharge. The 11 measures included in this submission have been endorsed since 2006 and results have been tied to hospital pay for reporting since 2007, and used in pay for performance and VBP since 2012.[61] Survey response rates for hospitals in our analysis range from 10% to 52%. The varying and often low response rates between hospitals led us to perform a sensitivity analysis of our findings with and without the patient experience measures (accessioned March 27, 2018, hospital compare. Data was used from the measure start date of 04/01/2014 till 03/31/2015).[61]

In addition to the four-domain based composite, several individual quality indicators were included in the analysis with a view to performing analysis comparable with published literature. These included the 2014 CMS Value Based Purchasing Total Performance Score (VBP-TPS), all-cause risk-adjusted 30-day readmission and 30-day mortality among adults, as well as risk-adjusted 30-day readmission and 30-day mortality for acute myocardial infarction (AMI), congestive heart failure (HF) and pneumonia (PN). The readmission measures, endorsed by the NQF, were obtained from the Hospital Inpatient Quality Reporting Program during calendar year 2014, and are available on the Hospital Compare website (http://www.hospitalcompare.hhs.gov). NQF endorsements have included the consideration of condition-specific readmission and mortality measures since April 2012 (http://www.qualityforum.org/ProjectDescription.aspx?projectID=73619).

### Measurement of hospital composite financial performance

We examined financial performance using data from CMS costs reports for the 2014 fiscal year and generated a continuous hospital composite financial performance score for each hospital based on a combination of financial measures.[62] Thus, our analyses considered multiple measures of financial health including operating profit or loss, net profit margin, return on total assets, cash flow margin, working capital, current ratio, days cash on hand, net asset position, equity financing, fixed asset financing, debt coverage, total debt ratio, long term debt ratio, salary ratio, total asset turnover, average operating margin and average total margin. These indicators were used to create a composite financial performance score. The CMS cost report data was obtained from the NYSDOH, although comparable data is publicly available from CMS. NYSDOH data were used due to ease of access.

### Hospital characteristics and covariates

To describe the hospitals studied and to adjust for potential confounding that may influence the financial performance of hospitals, the following hospital characteristics were utilized: teaching status, bed count, proportion of discharges with Medicare as a payer (Percent-Medicare), proportion of discharges with Medicaid as a payer (Percent-Medicaid), and rural versus urban geography.

Consistent with other research all hospitals were placed into one of three categories based on their response to the American Hospital Association (AHA) survey: major teaching hospitals (those that are members of the Council of Teaching Hospitals [COTH]), minor teaching hospitals (non-COTH members that had a medical school affiliation reported to the American Medical Association), and nonteaching hospitals (all other institutions) [63, 64]. Bed count assesses the number of short-term acute beds in the hospital, whether staffed or not, obtained from the AHA Annual Survey of Hospitals (Retrieved March 4^th^, 2016; https://www.ahadataviewer.com/quickreport/). Annual Medicare caseload was defined as the proportion of Medicare discharges divided by the total number of discharges, based on 2014 SPARCS data. Similarly, annual Medicaid caseload was defined as the proportion of Medicaid discharges divided by the total number of discharges based on 2014 SPARCS data. A hospital was considered urban if it was located in a metropolitan statistical area considered nonurban otherwise. This information was obtained from the AHA Annual Survey of Hospitals (Retrieved March 4^th^, 2016; https://www.ahadataviewer.com/quickreport/).

### Statistical analyses

Hospital level composite quality/safety performance scores and composite financial performance scores were developed. For each composite score, principal components analysis was used to synthesize the indicators simultaneously, loading weights were calculated based on indicator variance, and scores were standardized using the SAS Factor Procedure with varimax rotation. The number of factors to retain were determined based on the Scree Plots. The retained factors were used to calculate individual hospital composite scores by summing the individual hospital factor score weighted by the factor eigenvalue (variance explained) [65].

Several linear regression models were developed using the following permutations of dependent and independent variables, as well as with and without adjustment for percent Medicare and percent Medicaid, as follows:

$$Model\;1\;Quality=\alpha +\beta_{1}Financial+\beta_{2}BedSize+\beta_{3}Minor\;Teaching+\beta_{4}Non\;Teaching+\beta_{4}Urban+\beta_{5}\%Medicaid+\beta_{6}\%Medicare$$

$$Model\;2\;Quality=\alpha +\beta_{1}Financial+\beta_{2}BedSize+\beta_{3}Minor\;Teaching+\beta_{4}Non\;Teaching+\beta_{4}Urban$$

In order to compare the composite quality/safety performance scores calculated as described above, the following published quality metrics were also modeled: (1) CMS Value Based Purchasing Total Performance Score, (2) risk adjusted 30 day readmission for all patients, (3) risk adjusted 30 day readmission for AMI, (4) risk adjusted 30 day readmission for CHF, (5) risk adjusted 30 day readmission for PN, (6) risk adjusted 30 day mortality for AMI, and (7) risk adjusted 30 day mortality for CHF, (8) risk adjusted 30 day mortality for PN. Similarly, operating margin and total margin were modeled as independent financial variables to compare to the composite financial performance score.

All measures were standardized prior to regression analysis. Standardizing these coefficients allowed comparison of the relative importance of each coefficient in our regression models.[66] The strength of the coefficients based on standardized independent and dependent variables are internally comparable, and the strongest association is theoretically the one with the greatest total effect.[67] Model fit was assessed for influence and outliers in each model.

As an alternative to the standardized beta weights, regression tree models were developed using the Classification And Regression Trees (CART) methods.[68] Regression trees consist of recursive partitions of data into subsets according to ranges of ordered values of ordinal covariates or to subsets of values of categorical covariates which are as homogenous as possible with respect to the composite financial performance score. For all partitions, all observed covariates remain available even if they have been used earlier in the tree, so it is possible for a covariate to reappear at several points in a tree. Unlike traditional linear regression, covariates with similar information are kept in the process and assessed for every partition. CART ranks all covariates based on their contribution to the improvement in homogeneity (even if it does not appear in the tree). This is a measure of how “important” each covariate is based on explanatory power and, in the case of correlated covariates, based on their ability to perform as main splitting criterion. The process of building a regression tree requires a decision to stop partitioning the data. In this study, we stopped the trees when additional partitions did not improve homogeneity. Regression tree models for this study were developed using CART available in the Salford System’s Predictive Modeler v8.0. (https://www.salford-systems.com/products/cart)

## Results

Of the 214 non-federal acute care hospitals in New York in 2014, 109 (51%) were included in the principal component analysis. Reasons for exclusion were specialty facilities without medical or surgical beds (29), critical access facilities (18), recent closure (2), and ancillary facilities without independent financial information from a principal facility (56). The included hospitals account for 71% of inpatient discharges from non-Veteran’s Affairs (VA) NYS Hospitals in 2014.

### Composite financial performance score components

Principal component analysis of financial variables revealed seven factors with eigenvalues greater than one, accounting for 87 percent of the variance of the financial health subscale. The factors were interpreted as measuring profitability (38%), asset efficiency (13%), absolute size of assets (11%), debt coverage (9%), capital structure (7%), uncompensated care or unutilized income (5%), and growth (4%) (Table 1). The standardized composite financial performance scores for the 109 hospitals ranged from -3.70 to 3.05, Interquartile range (IQR) -0.45 to 0.38.

**Table 1:** Proportion of variance explained by principal components analysis of financial indicators, NYS hospitals, 2014.

Factors with key items listed	Proportion of variance explained	Cumulative variance explained
**Factor 1: Profitability** High Operating margin, net margin, return on assets, cash flow, current ratios, Low debt ratio, average payment period and asset turnover	38%	38%
**Factor 2: Asset Efficiency** High asset turnover, high margins, Low days cash on hand, current ratio, acid test ratio	13%	51%
**Factor 3: Absolute Size** High net assets, net assets	11%	62%
**Factor 4: Debt Coverage** High Capital expense ratio, current asset turnover, and capital expenditure growth rate, Low average payment period, debt ratio	9%	71%
**Factor 5: Capital Structure** High capital expenditure growth rate, total asset turnover, Low capital expense ratio	7%	78%
**Factor 6: Uncompensated Care/ Unutilized Income** Low days in patient accounts receivable	5%	83%
**Factor 7: Growth** High Capital Expenditure Growth Rate, Capital Expense Ratio	4%	87%

### Composite quality/patient safety performance components

Principal components analysis of quality variables revealed fourteen factors with eigenvalues greater than one, explaining a total of 77 percent of the variance of the quality/safety subscales. Based on analysis of scree plots, we narrowed the number of components to seven (Table 2), which explained 57 percent of the variance and had a very strong correlation (r = 0.91) with the 14-component summary score.

**Table 2:** Proportion of variance explained by principal components analysis of hospital quality indicators, New York State, 2014.

Factors with items listed in order	Proportion of variance explained	Cumulative variance explained
**Factor 1: Patient Experience of Care** Overall Rating (H-HSP-RATING), Care Transition (H-COMP-7), Willingness to Recommend (H-RCMND), Pain Management (H-COMP-4), Nurse Communication (H-COMP-1), Communication about Medicines (H-COMP-5), Responsiveness of Staff (H-COMP-3), Doctor Communication (H-COMP-2), Discharge Information (H-COMP-6), Cleanliness (H-CLEAN-HSP), Quietness (H-QUIET-HSP)	23%	23%
**Factor 2: Timeliness of care–Surgical Care Improvement** Prophylactic Antibiotic Received within 1 hour prior to surgical incision (SCIP-INF-1), Prophylactic antibiotic selection (SCIP-INF-2), Venous Thromboembolism Prophylaxis within 1 day of surgery (SCIP-VTE-2), Perioperative Beta Blocker if needed (SCIP-CARD-2), Influenza Immunization (IMM-2), Postoperative discontinuation of prophylactic antibiotics (SCIP-INF-3), Postoperative Urinary Catheter Removal (SCIP-INF-9)	10%	33%
**Factor 3: Timeliness of care–Stroke Care and DVT prevention** DVT prophylaxis within 1 day of admission or surgery (VTE-1), DVT treatment within 2 days of arrival for stroke patients (STK-1), DVT prophylaxis within 1 day of admission to ICU (VTE-2), Initial antibiotic selection for community acquired pneumonia patients (PN-6), Stroke patients assessed for rehabilitation services (STK-10)	7%	39%
**Factor 4: Timeliness of care–Emergency Department Delays** Median Time from ED Arrival to Admission for inpatient admissions (ED-1b), Median time to admission after decision to admit was made (ED-2b), Median Time from ED Arrival to ED Departure for Discharged Patients (OP-18b)	5%	45%
**Factor 5: Timeliness of care–Emergency Department Care** Median Time to pain medication for ED patients with bone fractures (OP-21), Percent of Patients that left ED without being seen (OP-22), Median Time from arrival to Diagnostic Evaluation (OP-20)	5%	49%
**Factor 6: Patient Safety** Accidental Puncture or Laceration (PSI-15), Wound Dehiscence (PSI-14), Postoperative Respiratory Failure Rate (PSI 11)	4%	53%
**Factor 7: Inpatient Mortality** Heart Failure Mortality Rate (IQI 16), Pneumonia Mortality Rate (IQI-20), Perioperative Hemorrhage or Hematoma (PSI-09), Stroke Patients receive antithrombotic therapy by end of hospital day 2 (STK-5).	4%	57%

The first component, interpreted as patient experience of care (23%), included all ten subscales derived from the HCAHPS survey. The second component, interpreted as timeliness in surgical care improvement (10%), included process of care subscales predominantly related to reducing poor surgical outcomes including cardiac, venous thromboembolism, and infections. The third component featured timeliness of stroke care and other prophylactic therapies (7%). The next two components were both related to emergency department (ED) process measures: factor four includes measures of ED delays following evaluation (5%) and factor five includes measures of ED quality, including timeliness of pain control and of evaluation. The sixth component included patient safety indicators (4%) and the seventh included inpatient mortality (6%). Additional analysis conducted without the patient experience measures found similar components and proportion of variance explained (43% for 5 factors, data not shown). The standardized composite quality/safety performance scores for the 109 hospitals ranged from -4.45 to 1.86, with an interquartile range (IQR) from -0.59 to 0.76. One facility was identified as a low outlier for both the standardized financial score (-3.70) and quality score (-4.45). To provide conservative estimates of association, this outlier was removed from the following regression analyses.

### Associations between hospital financial status and hospital quality of care

Stronger hospital financial standing, as measured using the composite financial performance score, was positively associated with better quality of care and service delivery as measured by the composite quality/safety performance score. (Table 3) Additionally, strong hospital financial standing also was associated with the CMS Value Based Purchasing Total Performance Score (VBP-TPS). The composite financial performance score was negatively associated with hospital wide 30-day readmission and 30-day readmission for heart failure and pneumonia, along with 30-day mortality from acute myocardial infarction (Table 3).

**Table 3:** Relationship between New York State hospital financial indicators and quality outcomes, without adjustment for percent medicare and percent medicaid (model1).

	Composite Financial Performance Score		Operating Margin		Total Margin	
	Parameter	p-value	Parameter	p-value	Parameter	p-value
Composite Quality/Safety Performance Score	***0*.*340***	***<0*.*001***	***0*.*215***	***0*.*02***	***0*.*212***	***0*.*02***
CMS Value Based Purchasing Total Performance Score	***0*.*277***	***0*.*002***	***0*.*218***	***0*.*02***	0.165	0.07
30 Day Readmissions for All Outcomes	***-0*.*236***	***0*.*013***	0.132	0.16	0.069	0.47
30 Day Readmissions for Congestive Heart Failure	***-0*.*229***	***0*.*02***	0.179	0.06	0.113	0.25
30 Day Readmissions for Pneumonia	***-0*.*209***	***0*.*033***	***0*.*214***	***0*.*03***	0.184	0.06
30 Day Readmissions for Acute Myocardial Infarction	-0.034	0.74	0.006	0.96	-0.013	0.91
30 Day Mortality for Acute Myocardial Infarction	***-0*.*211***	***0*.*027***	0.041	0.67	0.104	0.27

All parameters are standardized, and all models were adjusted for Bed Size, Teaching Hospital Status, and Urban/Rural Status. One outlier hospital excluded.

Overall, adjustment for Percent-Medicare coverage and Percent-Medicaid coverage attenuated the associations (Table 4). The composite quality/safety performance score, VBP-TPS and 30-day readmission for CHF remained statistically significant after adjustment for percent Medicaid and Medicare. The association between the composite financial performance score and a composite quality/safety performance score without patient experience measures was weaker (unadjusted for Medicare and Medicaid coverage: 0.169, p = 0.09; adjusted for Medicare and Medicaid coverage: 0.171, p = 0.12).

**Table 4:** Relationship between New York State hospital financial indicators and quality outcomes, with adjustment for percent medicare and percent medicaid (model2).

	Composite Financial Performance Score		Operating Margin		Total Margin	
	Parameter	p-value	Parameter	p-value	Parameter	p-value
Composite Quality/Safety Performance Score	***0*.*239***	***0*.*01***	0.106	0.25	0.112	0.23
CMS Value Based Purchasing Total Performance Score	***0*.*215***	***0*.*03***	**0.179**	**0.05**	0.098	0.29
30 Day Readmissions for All Outcomes	-0.162	0.10	0.044	0.64	-0.014	0.88
30 Day Readmissions for Congestive Heart Failure	***-0*.*230***	***0*.*02***	0.129	0.18	0.088	0.36
30 Day Readmissions for Pneumonia	-0.177	0.09	0.160	0.11	0.149	0.14
30 Day Readmissions for Acute Myocardial Infarction	-0.013	0.91	-0.023	0.84	-0.043	0.71
30 Day Mortality for Acute Myocardial Infarction	-0.162	0.12	0.004	0.97	0.048	0.62

All parameters are standardized, and all models were adjusted for Bed Size, Teaching Hospital Status, Urban/Rural Status, %Medicaid, and %Medicare. One outlier hospital excluded.

The composite quality/safety performance score regressed against the composite financial performance score with adjustment for percent Medicare and percent Medicaid had the best fit of all models, with an R-square of 0.29, p<0.0001. The model correlating VBP-TPS with the composite score including percent Medicare and percent Medicaid had an R-square of 0.27, p<0.0001. In the model of the composite quality score regressed against the composite financial without adjustment for percent Medicare and percent Medicaid the R-square was 0.21, p<0.0001.

Both operating margin (p = 0.02) and total margin (p = 0.02) demonstrated a statistically significant association with the composite quality score when the model was not adjusted for percent Medicare and percent Medicaid. With adjustment for percent Medicare and percent Medicaid, operating margin and total margin were not significantly associated with any of the quality metrics, except for one instance with operating margin associated with total VBP-TPS (Table 4). None of the financial measures used demonstrated a significant correlation with adjusted 30-day mortality for pneumonia and for congestive heart failure (data not shown).

### Decision-tree analyses

The results from regression tree models supported the findings from the traditional weighted linear regression models. The composite financial performance scores outperformed the total margin and the operating margin in predicting quality of care in NYS acute care hospitals. The composite financial performance score contributed with the largest reduction in mean squares in a regression tree model. The operating revenue margins contributed with a reduction less than half that of the composite financial performance score and the total margins was approximately 10% that of the composite financial score. This ranking remained similar when other predictors were added to the regression tree model. As more covariates were introduced in the regression tree model, the total margin and the operating revenue margin ended up as the weakest predictors of quality of care. However, the composite financial performance score had a similar performance as the total margin and clearly outperformed the operating margin when predicting the composite quality/safety performance score. The composite financial performance score contributed 83% reduction in mean squares compared to total margin and both measures clearly outperformed the operating margin which only contributed 32% reduction in mean squares. All three measures showed weak associations with the readmission measure.

## Discussion

Our analyses found strong evidence, as hypothesized, that financially stable hospitals have better patient experience, lower readmission rates, and show evidence of decreased risk of adverse patient quality and safety outcomes for both medical and surgical patients. Hospitals that are better off financially can maintain highly reliable systems and provide ongoing resources for quality improvement, as measured predominantly by patient experiences and better performance on process of care initiatives, while financially distressed facilities struggle in these categories. These superior outcomes in financially stable hospitals persisted after adjusting for public payer caseload and hospital characteristics, suggesting that underlying qualities of financially well-off facilities lead to medical and surgical care that is superior. A small number of studies have suggested a limited association between improved hospital financial performance and improved quality of care and patient safety in specific scenarios.[14, 17, 18, 48, 52] We improve on previous cross-sectional snapshots by developing financial and quality/safety composite measures that have improved predictive validity. The results suggest that money does matter.

In studying this relationship, we also recognize that measurement matters. The strength of the relationship between finances and quality in this report varies across the indicators used in these regression analyses. Financial health and quality/patient safety are complicated concepts that can be measured along many dimensions. Challenges arise when attempting to find elusive indicators for abstract, broad, and complicated measures, such as the financial health of organizations and/or quality of care of health facilities. Financial health can be measured considering capital structure, cost, profitability, liquidity and efficiency; while patient safety/quality care can range from hospital regulations adherence to patient perspectives on care.[62] The findings from previous studies on this topic are equivocal and have varying limitations [14, 25, 45–47, 50, 69–75]. This report attempts to overcome prior limitations related to measurement by integrating a broader spectrum of existing data routinely collected.

All measures were standardized prior to regression analysis. With standardization, the interpretation of the regression coefficients is the standard deviation change in the dependent variables per standard deviation change in independent variable. This technique preserves internal validity, but the standardized coefficients are only generalizable to other populations with similar variable distributions. Standardized coefficients also facilitate comparison between equations that use the same independent variable set.[66, 67] When comparing the various financial indicators and utilizing the model without adjustment for percent Medicare and Medicaid, the composite quality/safety performance score had the largest strength of effect followed by VBP-TPS and then various subsets of 30 day readmissions. Thirty-day mortality for acute myocardial infarction was also found to be significantly associated with financial health using this model, however none of the other indicators of mortality were significant in either model. The same pattern held when percent Medicare and percent Medicaid were included in the model, though strength was attenuated and fewer associations were significant.

When hospitals are compared to one another based on patient outcomes, concerns inevitably arise about risk-adjustment and statistical heterogeneity due to small numerators. To improve measurement, intermediate process and performance metrics have been added to the measure sets, raising concern of whether these measures appropriately inform meaningful health outcomes. [76] While there is modest evidence connecting many surrogate endpoints, such as risk-factor control or care processes, these metrics may be chosen because they are easy to access and measure, rather than being meaningful, patient-centered outcomes [76, 77]. With payment at stake, clinicians and health organizations may feel compelled to engage in gaming, in over-testing and overtreatment, or in devoting disproportionate effort to patients that improve these surrogate endpoints rather than focusing on those at highest risk [76, 77]. Furthermore, the availability and influence of these markers interferes with opportunities to establish more thoughtful interventions and individualized approaches to clinical complications such as social determinants and multimorbidity. [76, 78–82] We attempted to address these concerns by creating and using global risk measures representing both financial health and quality of care, as decisions for entire hospitals and health systems often rely on hospital level indicators. Global measures are more robust and are preferable to individual risk factors, as they are more likely to indicate highly reliable organizations by reducing the influence of gaming and interventions focused on improvement of individual metrics [76, 83, 84].

Variables chosen for adjustment in our models are well chronicled in the literature. It is well documented that greater dependence on government payers, such as Medicare and Medicaid are associated with a higher probability of financial distress because these payers typically do not pay the average full cost of care.[85–91] The analyses were adjusted for teaching hospital status as prior studies associated teaching hospitals with lower financial performance, considering they often support more labor-intensive staff and offer a wide array of costly medical services. The sheer size of a hospital, measured by the number of beds, allows a hospital to withstand costly outliers which could more likely have adverse effects on smaller facilities.[92] The mixture of operational and market factors influencing the financial condition of hospitals differently in urban versus non-urban areas is well documented.[19] Non-urban hospitals tend to be smaller and offer fewer services than urban hospitals. Finally, the outcome-based metrics used for the quality composite score were all based on published risk-adjustment methodology.[93, 94]

### Policy implications

Federal and State policy also matters as deficits in the quality of care can be systemic, requiring systems level modifications to produce the desired changes and results. As policy makers consider action to achieve the triple aim, the interrelatedness of cutting healthcare costs and achieving quality needs to be addressed, particularly as it affects the ability of fiscally distressed facilities serving vulnerable patients to engage in quality improvement.

This study has policy implications for the millions of patients who gained Medicaid coverage beginning in 2014 from the Affordable Care Act and for the future of the Medicaid program, in general. The attenuation of the association observed here when controlling for public payer are consistent with previous studies that found that hospitals with high Medicaid case-mix had worse quality of care than other hospitals.[56, 95–100] Research on nursing homes also suggest a link between lower Medicaid reimbursement levels and lower quality.[101–104] Despite this association, powerful evidence has been published suggesting that Medicaid has a positive impact on access to care, financial security, and self-reported health.[105, 106] At the same time, under the veil of deficit reduction, future expected cutbacks could lead to reduced access to high quality care. Our findings suggest that any cost-cutting efforts by Medicare, Medicaid, and private payers needs to be carefully designed and managed so that patient safety and patient centered care are not compromised.

Since our analysis was performed with hospital level data, we cannot examine variation in individual patient care within each hospital. Patient level analysis would be relevant if the disparity in quality that arises from financial distress contributed to disparities in outcomes that have been observed for vulnerable patient populations, including older and poorer patients, those covered by Medicaid or Medicare, those with complex comorbidities, and medically disadvantaged groups such as racial and gender minorities.[107, 108] [109–114] Evaluating performance on the hospital level of analysis, however, is relevant since systems and policy decisions are most commonly determined either at the hospital, state, or federal level [57]. Further, there is no current source of individual level data on patient experience of care, which the analysis presented here confirms accounts for the most variability across the hospital healthcare system. The results presented here represents the state of affairs in New York prior to the launch of the Delivery System Reform Incentive Payment (DSRIP) Program funded by the Section 1115 Demonstration Waiver [115]. Repeating these analyses at the end of New York’s five-year demonstration will identify if systemic changes have reduced the chasms in quality of care, or made them deeper.[116]

Our findings support the notion that hospitals under greater financial distress have less favorable patient experience of care, higher readmission rates, and increased risk of adverse patient quality and safety outcomes for both medical and surgical patients. These substandard outcomes in financially distressed hospitals persisted after adjusting for public payer caseload and hospital characteristics. This suggests that underlying qualities of poorer facilities can lead to medical and surgical care that is inferior as well as an inferior experience for patients. This study provides composite measures that optimized the estimated correlation between financial status and quality/patient safety outcomes. These findings suggest that it is imperative to address financial disparities when incentivizing health care quality through value-based purchasing in order to ensure financial stability and quality of care in safety net facilities.

## References

[1] Loubeau PR, Jantzen R. U.S. hospital bond ratings in the managed care era. J Health Care Finance. 2005;31(3):41–51. Epub 2005/08/06. . 16080414

[2] Kim TH, McCue MJ. Association of market, operational, and financial factors with nonprofit hospitals' capital investment. Inquiry. 2008;45(2):215–31. Epub 2008/09/05. 10.5034/inquiryjrnl_45.02.215 . 18767385

[3] Commission J. <Health care at the crossroads: guiding principles for the development of the hospital of the future.pdf>. 2009.

[4] Bazzoli GJ, Clement JP, Lindrooth RC, Chen H-F, Aydede SK, Braun BI, et al Hospital financial condition and operational decisions related to the quality of hospital care. Medical Care Research and Review. 2007;64(2):148–68. 10.1177/1077558706298289 17406018

[5] Encinosa WE, Bernard DM. Hospital finances and patient safety outcomes. Inquiry. 2005;42(1):60–72. Epub 2005/07/15. 10.5034/inquiryjrnl_42.1.60 . 16013586

[6] Mueller SK, Lipsitz S, Hicks LS. Impact of hospital teaching intensity on quality of care and patient outcomes. Med Care. 2013;51(7):567–74. Epub 2013/04/23. 10.1097/MLR.0b013e3182902151 . 23604017

[7] Dong GN. Performing well in financial management and quality of care: evidence from hospital process measures for treatment of cardiovascular disease. BMC Health Serv Res. 2015;15:45 Epub 2015/02/02. 10.1186/s12913-015-0690-x 25638252 PMC4345031

[8] Hoehn RS, Wima K, Vestal MA, Weilage DJ, Hanseman DJ, Abbott DE, et al Effect of Hospital Safety-Net Burden on Cost and Outcomes After Surgery. JAMA Surg. 2016;151(2):120–8. Epub 2015/10/16. 10.1001/jamasurg.2015.3209 . 26466334

[9] Jha AK, Orav EJ, Epstein AM. The effect of financial incentives on hospitals that serve poor patients. Ann Intern Med. 2010;153(5):299–306. Epub 2010/09/08. 10.7326/0003-4819-153-5-201009070-00004 . 20820039

[10] Smith RB, Dynan L, Fairbrother G, Chabi G, Simpson L. Medicaid, hospital financial stress, and the incidence of adverse medical events for children. Health Serv Res. 2012;47(4):1621–41. Epub 2012/02/23. 10.1111/j.1475-6773.2012.01385.x 22353008 PMC3401402

[11] Weissman JS, Vogeli C, Levy DE. The quality of hospital care for Medicaid and private pay patients. Med Care. 2013;51(5):389–95. Epub 2013/01/30. 10.1097/MLR.0b013e31827fef95 . 23358385

[12] Platt HD, Platt MB. Predicting corporate financial distress: reflections on choice-based sample bias. Journal of Economics and Finance. 2002;26(2):184–99.

[13] Ly DP, Jha AK, Epstein AM. The association between hospital margins, quality of care, and closure or other change in operating status. J Gen Intern Med. 2011;26(11):1291–6. Epub 2011/08/13. 10.1007/s11606-011-1815-5 21837374 PMC3208470

[14] Bazzoli GJ, Chen HF, Zhao M, Lindrooth RC. Hospital financial condition and the quality of patient care. Health Econ. 2008;17(8):977–95. Epub 2007/12/25. 10.1002/hec.1311 . 18157911

[15] Volpp KG, Konetzka RT, Zhu J, Parsons L, Peterson E. Effect of cuts in Medicare reimbursement on process and outcome of care for acute myocardial infarction patients. Circulation. 2005;112(15):2268–75. Epub 2005/10/06. 10.1161/CIRCULATIONAHA.105.534164 . 16203913

[16] Krumholz HM, Lin Z, Keenan PS, Chen J, Ross JS, Drye EE, et al Relationship between hospital readmission and mortality rates for patients hospitalized with acute myocardial infarction, heart failure, or pneumonia. Jama. 2013;309(6):587–93. Epub 2013/02/14. 10.1001/jama.2013.333 23403683 PMC3621028

[17] Encinosa WE, Bernard DM. Hospital finances and patient safety outcomes. INQUIRY: The Journal of Health Care Organization, Provision, and Financing. 2005;42(1):60–72. 10.5034/inquiryjrnl_42.1.60 16013586

[18] Ly DP, Jha AK, Epstein AM. The association between hospital margins, quality of care, and closure or other change in operating status. Journal of general internal medicine. 2011;26(11):1291 10.1007/s11606-011-1815-5 21837374 PMC3208470

[19] Brecher C, Nesbitt S. Factors associated with variation in financial condition among voluntary hospitals. Health Serv Res. 1985;20(3):267–300. Epub 1985/08/01. 4019212 PMC1068881

[20] Kim TH. Factors associated with financial distress of nonprofit hospitals. Health Care Manag (Frederick). 2010;29(1):52–62. Epub 2010/02/11. 10.1097/HCM.0b013e3181cca2c5 . 20145468

[21] Leatherman S, Berwick D, Iles D, Lewin LS, Davidoff F, Nolan T, et al The business case for quality: case studies and an analysis. Health affairs (Project Hope). 2003;22(2):17–30. Epub 2003/04/04. . 12674405 10.1377/hlthaff.22.2.17

[22] Marshall MN, Shekelle PG, Davies HT, Smith PC. Public reporting on quality in the United States and the United Kingdom. Health affairs (Project Hope). 2003;22(3):134–48. Epub 2003/05/22. 10.1377/hlthaff.22.3.134 . 12757278

[23] Traska MR. Will operating margins limit access to capital? Hospitals. 1988;62(2):38–44. Epub 1988/01/20. . 3335370

[24] Sherlock DB. Indigent care in rational markets. Inquiry. 1986;23(3):261–7. Epub 1986/01/01. . 2944835

[25] Lindrooth RC, Bazzoli GJ, Clement J. The effect of reimbursement on the intensity of hospital services. Southern Economic Journal. 2007:575–87.

[26] Duffy SQ, Friedman B. Hospitals with chronic financial losses: what came next? Health affairs (Project Hope). 1993;12(2):151–63. Epub 1993/01/01. . 8375810 10.1377/hlthaff.12.2.151

[27] Burstin HR, Lipsitz SR, Udvarhelyi IS, Brennan TA. The effect of hospital financial characteristics on quality of care. Jama. 1993;270(7):845–9. Epub 1993/08/18. . 8340984

[28] Mouch CA, Regenbogen SE, Revels SL, Wong SL, Lemak CH, Morris AM. The quality of surgical care in safety net hospitals: a systematic review. Surgery. 2014;155(5):826–38. Epub 2014/05/03. 10.1016/j.surg.2013.12.006 . 24787109

[29] Martsolf GR, Auerbach D, Benevent R, Stocks C, Jiang HJ, Pearson ML, et al Examining the value of inpatient nurse staffing: an assessment of quality and patient care costs. Med Care. 2014;52(11):982–8. Epub 2014/10/12. 10.1097/MLR.0000000000000248 . 25304017

[30] Dranove D, White WD. Medicaid-dependent hospitals and their patients: how have they fared? Health Serv Res. 1998;33(2 Pt 1):163–85. Epub 1998/06/10. 9618666 PMC1070259

[31] Langa KM, Sussman EJ. The effect of cost-containment policies on rates of coronary revascularization in California. The New England journal of medicine. 1993;329(24):1784–9. Epub 1993/12/09. 10.1056/NEJM199312093292407 . 8232488

[32] Bazzoli GJ, Lindrooth RC, Hasnain-Wynia R, Needleman J. The Balanced Budget Act of 1997 and U.S. hospital operations. Inquiry. 2004;41(4):401–17. Epub 2005/04/20. 10.5034/inquiryjrnl_41.4.401 . 15835599

[33] Silow-Carroll S, Alteras T, Meyer JA. Hospital quality improvement: strategies and lessons from US hospitals. New York: The Commonwealth Fund 2007.

[34] Banks DA, Paterson M, Wendel J. Uncompensated hospital care: charitable mission or profitable business decision? Health Econ. 1997;6(2):133–43. Epub 1997/03/01. . 9158966 10.1002/(sici)1099-1050(199703)6:2<133::aid-hec252>3.0.co;2-x

[35] Hibbard JH, Stockard J, Tusler M. Hospital performance reports: impact on quality, market share, and reputation. Health affairs (Project Hope). 2005;24(4):1150–60. Epub 2005/07/14. 10.1377/hlthaff.24.4.1150 . 16012155

[36] Swensen SJ, Dilling JA, Mc Carty PM, Bolton JW, Harper CM Jr. The business case for health-care quality improvement. J Patient Saf. 2013;9(1):44–52. Epub 2013/02/23. 10.1097/PTS.0b013e3182753e33 . 23429226

[37] Casalino LP, Elster A, Eisenberg A, Lewis E, Montgomery J, Ramos D. Will pay-for-performance and quality reporting affect health care disparities? Health affairs (Project Hope). 2007;26(3):w405–14. Epub 2007/04/12. 10.1377/hlthaff.26.3.w405 . 17426053

[38] Chien AT, Chin MH, Davis AM, Casalino LP. Pay for performance, public reporting, and racial disparities in health care: how are programs being designed? Medical care research and review: MCRR. 2007;64(5 Suppl):283s–304s. Epub 2007/10/19. 10.1177/1077558707305426 . 17881629

[39] Epstein AM, Lee TH, Hamel MB. Paying physicians for high-quality care. The New England journal of medicine. 2004;350(4):406–10. Epub 2004/01/23. 10.1056/NEJMsb035374 . 14736934

[40] Laschober M, Maxfield M, Felt-Lisk S, Miranda DJ. Hospital response to public reporting of quality indicators. Health Care Financ Rev. 2007;28(3):61–76. Epub 2007/07/25. 17645156 PMC4194994

[41] Werner RM, Goldman LE, Dudley RA. Comparison of change in quality of care between safety-net and non-safety-net hospitals. Jama. 2008;299(18):2180–7. Epub 2008/05/15. 10.1001/jama.299.18.2180 . 18477785

[42] Kim TH. Factors associated with financial distress of nonprofit hospitals. The health care manager. 2010;29(1):52–62. 10.1097/HCM.0b013e3181cca2c5 20145468

[43] Kim TH, McCue MJ. Association of market, operational, and financial factors with nonprofit hospitals' capital investment. INQUIRY: The Journal of Health Care Organization, Provision, and Financing. 2008;45(2):215–31. 10.5034/inquiryjrnl_45.02.215 18767385

[44] Swensen SJ, Dilling JA, Mc Carty PM, Bolton JW, Harper CM Jr. The business case for health-care quality improvement. Journal of patient safety. 2013;9(1):44–52. 10.1097/PTS.0b013e3182753e33 23429226

[45] Volpp KG, Konetzka RT, Zhu J, Parsons L, Peterson E. Effect of cuts in Medicare reimbursement on process and outcome of care for acute myocardial infarction patients. Circulation. 2005;112(15):2268–75. 10.1161/CIRCULATIONAHA.105.534164 16203913

[46] Hadley J, Zuckerman S, Feder J. Profits and fiscal pressure in the prospective payment system: their impacts on hospitals. Inquiry. 1989;26(3):354–65. Epub 1989/01/01. . 2529213

[47] Shen YC. The effect of financial pressure on the quality of care in hospitals. J Health Econ. 2003;22(2):243–69. Epub 2003/02/28. 10.1016/S0167-6296(02)00124-8 . 12606145

[48] Nguyen OK, Halm EA, Makam AN. Relationship between hospital financial performance and publicly reported outcomes. Journal of hospital medicine. 2016;11(7):481–8. 10.1002/jhm.2570 26929094 PMC5362822

[49] Hoehn RS, Wima K, Vestal MA, Weilage DJ, Hanseman DJ, Abbott DE, et al Effect of hospital safety-net burden on cost and outcomes after surgery. JAMA surgery. 2016;151(2):120–8. 10.1001/jamasurg.2015.3209 26466334

[50] Clement JP, Lindrooth RC, Chukmaitov AS, Chen H-F. Does the patient's payer matter in hospital patient safety?: a study of urban hospitals. Medical care. 2007;45(2):131–8. 10.1097/01.mlr.0000244636.54588.2b 17224775

[51] Duffy SQ, Friedman B. Hospitals with chronic financial losses: what came next? Health Affairs. 1993;12(2):151–63. 8375810 10.1377/hlthaff.12.2.151

[52] Nguyen OK, Halm EA, Makam AN. Relationship between hospital financial performance and publicly reported outcomes. J Hosp Med. 2016;11(7):481–8. Epub 2016/03/02. 10.1002/jhm.2570 26929094 PMC5362822

[53] Jha AK, Li Z, Orav EJ, Epstein AM. Care in U.S. hospitals—the Hospital Quality Alliance program. The New England journal of medicine. 2005;353(3):265–74. Epub 2005/07/22. 10.1056/NEJMsa051249 . 16034012

[54] Jha AK, Orav EJ, Epstein AM. The effect of financial incentives on hospitals that serve poor patients. Annals of internal medicine. 2010;153(5):299–306. 10.7326/0003-4819-153-5-201009070-00004 20820039

[55] Mueller SK, Lipsitz S, Hicks LS. Impact of hospital teaching intensity on quality of care and patient outcomes. Medical care. 2013;51(7):567–74. 10.1097/MLR.0b013e3182902151 23604017

[56] Goldman LE, Vittinghoff E, Dudley RA. Quality of care in hospitals with a high percent of Medicaid patients. Med Care. 2007;45(6):579–83. Epub 2007/05/23. 10.1097/MLR.0b013e318041f723 . 17515786

[57] Cefalu MS, Elliott MN, Setodji CM, Cleary PD, Hays RD. Hospital quality indicators are not unidimensional: A reanalysis of Lieberthal and Comer. Health services research. 2019;54(2):502–8. 10.1111/1475-6773.13056 30259508 PMC6407350

[58] Indicators AQ. Guide to inpatient quality indicators 2002 [cited 2018 09/12/2018]. Available from: https://www.ahrq.gov/downloads/pub/inpatqi/iqi_guide.pdf.

[59] Health NYSDo. Hospital Inpatient Discharges (SPARCS De-Identified) Downloadable File: 2014. February 5, 2016 ed. http://health.data.ny.gov/Health/Hospital-Inpatient-Discharges-SPARCS-De-Identified/rmwa-zns42014.

[60] Services CfMM. HCAHPS Fact Sheet 2017 [updated November 2017]. Available from: http://www.hcahpsonline.org/globalassets/hcahps/facts/hcahps_fact_sheet_november_2017a.pdf.

[61] Forum NQ. National Quality Forum Endorsed Measures for Person and Family Centered Care.pdf [Draft Report]. 2014 [updated September 5, 2014]. Available from: http://www.qualityforum.org/projects/person_family_centered_care/.

[62] Gapenski LC, Reiter KL. Healthcare finance: an introduction to accounting and financial management: Health Administration Press Chicago, IL; 2008.

[63] Rosenthal GE, Harper DL, Quinn LM, Cooper GS. Severity-adjusted mortality and length of stay in teaching and nonteaching hospitals: results of a regional study. Jama. 1997;278(6):485–90. 9256223

[64] Allison JJ, Kiefe CI, Weissman NW, Person SD, Rousculp M, Canto JG, et al Relationship of hospital teaching status with quality of care and mortality for Medicare patients with acute MI. Jama. 2000;284(10):1256–62. 10.1001/jama.284.10.1256 10979112

[65] Lieberthal RD, Comer DM. What are the characteristics that explain hospital quality? A longitudinal pridit approach. Risk Management and Insurance Review. 2014;17(1):17–35.

[66] Freedman DA. Statistical models: theory and practice: cambridge university press; 2009.

[67] Pedhazur E. Multiple regression in behavioral research: Explanation and prediction. Third ed Thomson Learning Inc: Christopher P. Klein; 1997.

[68] Breiman L. Classification and regression trees: Routledge; 2017.

[69] Feder J, Hadley J, Zuckerman S. How did Medicare's prospective payment system affect hospitals? New England Journal of Medicine. 1987;317(14):867–73. 10.1056/NEJM198710013171405 3306387

[70] Hadley J, Zuckerman S, Feder J. Profits and fiscal pressure in the prospective payment system: Their impacts on hospitals. Inquiry. 1989:354–65. 2529213

[71] Shen Y-C. The effect of financial pressure on the quality of care in hospitals. Journal of health economics. 2003;22(2):243–69. 10.1016/S0167-6296(02)00124-8 12606145

[72] Volpp K, Buckley E. The effect of increases in HMO penetration and changes in payer mix on in-hospital mortality and treatment patterns for acute myocardial infarction. The American journal of managed care. 2004;10(7 Pt 2):505–12. 15298238

[73] Feder J, Hadley J, Zuckerman S. How did Medicare's prospective payment system affect hospitals? The New England journal of medicine. 1987;317(14):867–73. Epub 1987/10/01. 10.1056/NEJM198710013171405 . 3306387

[74] Clement JP, Lindrooth RC, Chukmaitov AS, Chen HF. Does the patient's payer matter in hospital patient safety?: a study of urban hospitals. Med Care. 2007;45(2):131–8. Epub 2007/01/17. 10.1097/01.mlr.0000244636.54588.2b . 17224775

[75] Volpp KG, Buckley E. The effect of increases in HMO penetration and changes in payer mix on in-hospital mortality and treatment patterns for acute myocardial infarction. Am J Manag Care. 2004;10(7 Pt 2):505–12. Epub 2004/08/10. . 15298238

[76] Saver BG, Martin SA, Adler RN, Candib LM, Deligiannidis KE, Golding J, et al Care that Matters: Quality Measurement and Health Care. PLOS Medicine. 2015;12(11):e1001902 10.1371/journal.pmed.1001902 26574742 PMC4648519

[77] Glasziou PP, Buchan H, Del Mar C, Doust J, Harris M, Knight R, et al When financial incentives do more good than harm: a checklist. BMJ (Clinical research ed). 2012;345:e5047 Epub 2012/08/16. 10.1136/bmj.e5047 . 22893568

[78] Eddy DM, Adler J, Patterson B, Lucas D, Smith KA, Morris M. Individualized guidelines: the potential for increasing quality and reducing costs. Ann Intern Med. 2011;154(9):627–34. Epub 2011/05/04. 10.7326/0003-4819-154-9-201105030-00008 . 21536939

[79] Guthrie B, Payne K, Alderson P, McMurdo ME, Mercer SW. Adapting clinical guidelines to take account of multimorbidity. BMJ (Clinical research ed). 2012;345:e6341 Epub 2012/10/06. 10.1136/bmj.e6341 . 23036829

[80] McGlynn EA, Schneider EC, Kerr EA. Reimagining quality measurement. The New England journal of medicine. 2014;371(23):2150–3. Epub 2014/12/04. 10.1056/NEJMp1407883 . 25470693

[81] McShane M, Mitchell E. Person centred coordinated care: where does the QOF point us? BMJ (Clinical research ed). 2015;350:h2540 Epub 2015/06/13. 10.1136/bmj.h2540 . 26067130

[82] Loxterkamp D. Humanism in the time of metrics—an essay by David Loxterkamp. BMJ (Clinical research ed). 2013;347:f5539 Epub 2013/09/21. 10.1136/bmj.f5539 . 24052583

[83] Eddy DM, Adler J, Morris M. The ‘Global Outcomes Score’: A Quality Measure, Based On Health Outcomes, That Compares Current Care To A Target Level Of Care. Health Affairs. 2012;31(11):2441–50. 10.1377/hlthaff.2011.1274 23129674

[84] Eddy DM, Adler J, Morris M. The 'Global Outcomes Score': a quality measure, based on health outcomes, that compares current care to a target level of care. Health affairs (Project Hope). 2012;31(11):2441–50. Epub 2012/11/07. 10.1377/hlthaff.2011.1274 . 23129674

[85] Vogel WB, Langland-Orban B, Gapenski LC. Factors influencing high and low profitability among hospitals. Health Care Management Review. 1993;18(2):15–26. 8320103

[86] Dranove D, White WD. Medicaid-dependent hospitals and their patients: How have they fared? Health services research. 1998;33(2 Pt 1):163. 9618666 PMC1070259

[87] McCue MJ, Renn SC, Pillari GD. Factors affecting credit rating downgrades of hospital revenue bonds. Inquiry. 1990:242–54. 2145225

[88] Rosko MD. The supply of uncompensated care in Pennsylvania hospitals: motives and financial consequences. Health Care Management Review. 2004;29(3):229–39. 15357233 10.1097/00004010-200407000-00008

[89] Vogel WB, Langland-Orban B, Gapenski LC. Factors influencing high and low profitability among hospitals. Health Care Manage Rev. 1993;18(2):15–26. Epub 1993/01/01. . 8320103

[90] McCue MJ, Renn SC, Pillari GD. Factors affecting credit rating downgrades of hospital revenue bonds. Inquiry. 1990;27(3):242–54. Epub 1990/01/01. . 2145225

[91] Rosko MD. The supply of uncompensated care in Pennsylvania hospitals: motives and financial consequences. Health Care Manage Rev. 2004;29(3):229–39. Epub 2004/09/11. . 15357233 10.1097/00004010-200407000-00008

[92] McCue MJ. The use of cash flow to analyze financial distress in California hospitals. Journal of Healthcare Management. 1991;36(2):223. 10110408

[93] Davies SM, Geppert J, McClellan M, McDonald KM, Romano PS, Shojania KG. Refinement of the HCUP quality indicators. 2001. 20734520

[94] Davies SM, Geppert J, McClellan M, McDonald KM, Romano PS, Shojania KG. AHRQ Technical Reviews. Refinement of the HCUP Quality Indicators. Rockville (MD): Agency for Healthcare Research and Quality (US); 2001. 20734520

[95] Goldman LE, Vittinghoff E, Dudley RA. Quality of care in hospitals with a high percent of Medicaid patients. Medical care. 2007;45(6):579–83. 10.1097/MLR.0b013e318041f723 17515786

[96] Werner RM, Goldman LE, Dudley RA. Comparison of change in quality of care between safety-net and non–safety-net hospitals. Jama. 2008;299(18):2180–7. 10.1001/jama.299.18.2180 18477785

[97] Popescu I, Werner RM, Vaughan-Sarrazin MS, Cram P. Characteristics and outcomes of America’s lowest-performing hospitals: an analysis of acute myocardial infarction hospital care in the United States. Circulation: Cardiovascular Quality and Outcomes. 2009;2(3):221–7. 10.1161/CIRCOUTCOMES.108.813790 20031841 PMC5361404

[98] Landon BE, Schneider EC, Normand S-LT, Scholle SH, Pawlson LG, Epstein AM. Quality of care in Medicaid managed care and commercial health plans. JAMA. 2007;298(14):1674–81. 10.1001/jama.298.14.1674 17925519

[99] Popescu I, Werner RM, Vaughan-Sarrazin MS, Cram P. Characteristics and outcomes of America's lowest-performing hospitals: an analysis of acute myocardial infarction hospital care in the United States. Circulation Cardiovascular quality and outcomes. 2009;2(3):221–7. Epub 2009/12/25. 10.1161/CIRCOUTCOMES.108.813790 20031841 PMC5361404

[100] Landon BE, Schneider EC, Normand SL, Scholle SH, Pawlson LG, Epstein AM. Quality of care in Medicaid managed care and commercial health plans. Jama. 2007;298(14):1674–81. Epub 2007/10/11. 10.1001/jama.298.14.1674 . 17925519

[101] Mor V, Gruneir A, Feng Z, Grabowski DC, Intrator O, Zinn J. The effect of state policies on nursing home resident outcomes. Journal of the American Geriatrics Society. 2011;59(1):3–9. 10.1111/j.1532-5415.2010.03230.x 21198463 PMC3731756

[102] Grabowski DC, Gruber J, Angelelli JJ. Nursing home quality as a common good. The Review of Economics and Statistics. 2008;90(4):754–64. 10.1162/rest.90.4.754 20463859 PMC2867608

[103] Mor V, Gruneir A, Feng Z, Grabowski DC, Intrator O, Zinn J. The effect of state policies on nursing home resident outcomes. J Am Geriatr Soc. 2011;59(1):3–9. Epub 2011/01/05. 10.1111/j.1532-5415.2010.03230.x 21198463 PMC3731756

[104] Grabowski DC, Gruber J, Angelelli JJ. Nursing Home Quality as a Common Good. Rev Econ Stat. 2008;90(4):754–64. Epub 2008/11/01. 10.1162/rest.90.4.754 20463859 PMC2867608

[105] Baicker K, Finkelstein A. The effects of Medicaid coverage—learning from the Oregon experiment. New England Journal of Medicine. 2011;365(8):683–5. 10.1056/NEJMp1108222 21774703 PMC3321578

[106] Baicker K, Finkelstein A. The effects of Medicaid coverage—learning from the Oregon experiment. The New England journal of medicine. 2011;365(8):683–5. Epub 2011/07/22. 10.1056/NEJMp1108222 21774703 PMC3321578

[107] Gaskin DJ, Spencer CS, Richard P, Anderson GF, Powe NR, LaVeist TA. Do hospitals provide lower-quality care to minorities than to whites? Health Affairs. 2008;27(2):518–27. 10.1377/hlthaff.27.2.518 18332510

[108] Gaskin DJ, Spencer CS, Richard P, Anderson GF, Powe NR, Laveist TA. Do hospitals provide lower-quality care to minorities than to whites? Health affairs (Project Hope). 2008;27(2):518–27. Epub 2008/03/12. 10.1377/hlthaff.27.2.518 . 18332510

[109] Barnato AE, Lucas FL, Staiger D, Wennberg DE, Chandra A. Hospital-level racial disparities in acute myocardial infarction treatment and outcomes. Medical care. 2005;43(4):308 15778634 10.1097/01.mlr.0000156848.62086.06 PMC2121607

[110] Hasnain-Wynia R, Baker DW, Nerenz D, Feinglass J, Beal AC, Landrum MB, et al Disparities in health care are driven by where minority patients seek care: examination of the hospital quality alliance measures. Archives of Internal Medicine. 2007;167(12):1233–9. 10.1001/archinte.167.12.1233 17592095

[111] Smedley BD, Stith AY, Nelson AR. Institute of Medicine, Committee on Understanding and Eliminating Racial and Ethnic Disparities in Health Care Unequal treatment: confronting racial and ethnic disparities in healthcare. Washington, DC: National Academies Press; 2003. 25032386

[112] Barnato AE, Lucas FL, Staiger D, Wennberg DE, Chandra A. Hospital-level racial disparities in acute myocardial infarction treatment and outcomes. Med Care. 2005;43(4):308–19. Epub 2005/03/22. 15778634 10.1097/01.mlr.0000156848.62086.06 PMC2121607

[113] Hasnain-Wynia R, Baker DW, Nerenz D, Feinglass J, Beal AC, Landrum MB, et al Disparities in health care are driven by where minority patients seek care: examination of the hospital quality alliance measures. Arch Intern Med. 2007;167(12):1233–9. Epub 2007/06/27. 10.1001/archinte.167.12.1233 . 17592095

[114] Institute of Medicine Committee on U, Eliminating R, Ethnic Disparities in Health C. In: Smedley BD, Stith AY, Nelson AR, editors. Unequal Treatment: Confronting Racial and Ethnic Disparities in Health Care. Washington (DC): National Academies Press (US) Copyright 2002 by the National Academy of Sciences All rights reserved.; 2003. 25032386

[115] Roby DH, Louis CJ, Cole MMJ, Chau N, Wiefling B, Salsberry DC, et al Supporting transformation through delivery system reform incentive payment programs: lessons from New York state. 2018. 10.1215/03616878-4303527 29630709

[116] Corrigan JM. Crossing the quality chasm. Building A Better Delivery System. 2005.

